# Dysphagia assessment based on photoacoustic imaging: a pilot ex vivo and in vivo study in infant swine models

**DOI:** 10.1007/s44258-025-00062-6

**Published:** 2025-10-22

**Authors:** Yanda Cheng, Chuqin Huang, Robert W. Bing, Emily Zheng, Huijuan Zhang, Wenyao Xu, Christopher Mayerl, Rebecca German, Catriona M. Steele, Jonathan Lovell, Lin Zhang, Jun Xia

**Affiliations:** 1 Department of Biomedical Engineering, University at Buffalo, The State University of New York, Buffalo, NY USA; 2 Department of Computer Science and Engineering, University at Buffalo, The State University of New York, Buffalo, NY USA; 3 Department of Anatomy and Neurobiology, Northeast Ohio Medical University, Rootstown, OH USA; 4 Department of Biological Sciences, Northern Arizona University, Flagstaff, AZ USA; 5 Toronto Rehabilitation Institute, University Health Network, Toronto, ON Canada

**Keywords:** Photoacoustic imaging, Dysphagia, Swallow assessment, Ultrasound, Videofluoroscopy, Aspiration

## Abstract

Swallowing impairments, such as dysphagia, pose significant health risks, including aspiration pneumonia, especially in vulnerable populations like infants and the elderly. Traditional diagnostic methods like videofluoroscopy and Fiberoptic Endoscopic Evaluation of Swallowing have limitations, including radiation exposure and discomfort. This study explores the potential of photoacoustic imaging as a non-invasive alternative for detecting swallowing events. Utilizing a 10 mg/mL charcoal solution as a contrast agent, we conducted both ex-vivo and in-vivo experiments using pig models. The ex-vivo tests on pig cadavers validated the system’s ability in detecting charcoal flow in the airway. Subsequent in-vivo experiments on live pigs, conducted with synchronized videofluoroscopy, demonstrated photoacoustic’s potential in seeing the same structure as videofluoroscopy. Our preliminary investigation indicates that photoacoustic imaging could offer a safer, more accurate method for dysphagia assessment, particularly in pediatric settings.

**Graphical Abstract:**

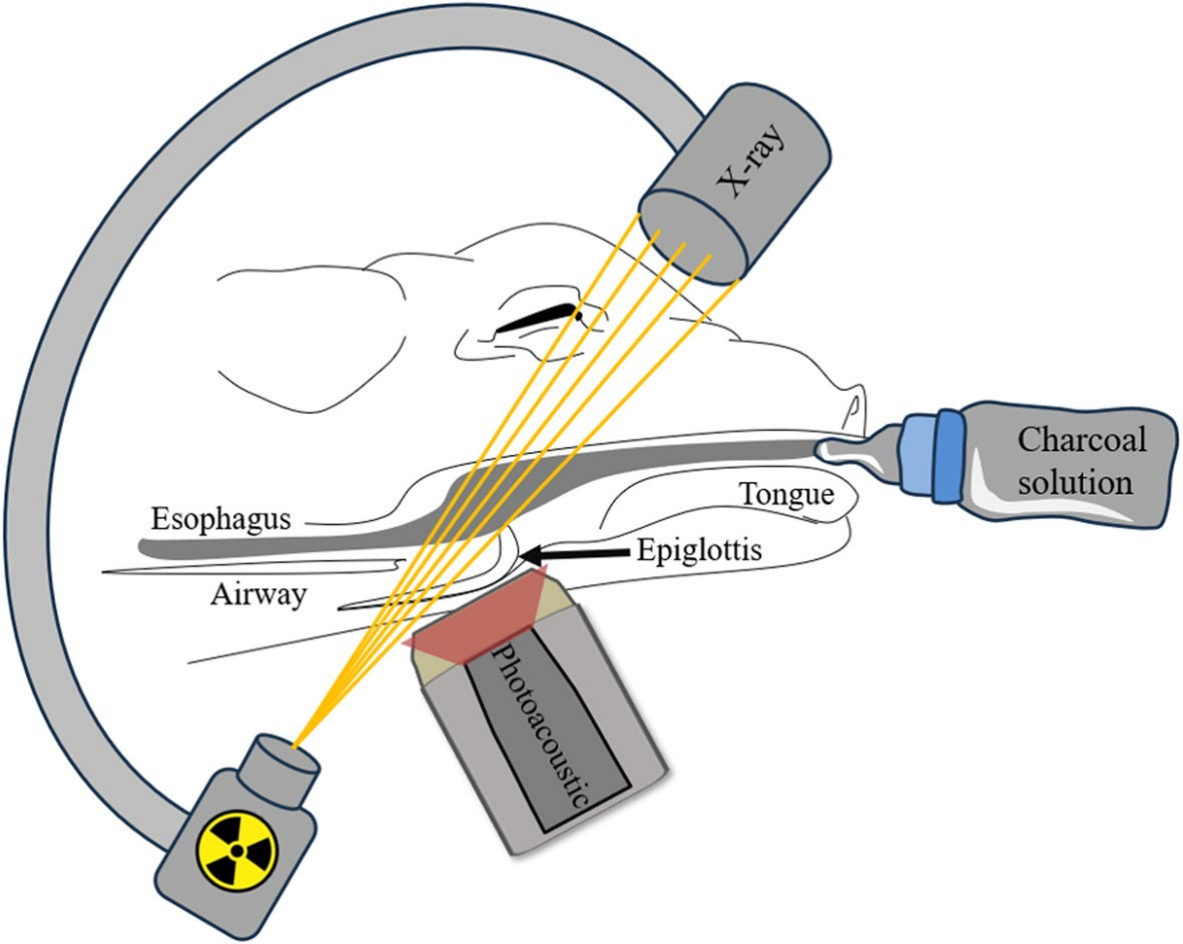

**Supplementary Information:**

The online version contains supplementary material available at 10.1007/s44258-025-00062-6.

## Highlights

• Dual-model imaging system includes photoacoustic imaging with videofluoroscopy, enhanced by a charcoal contrast agent, successfully captures swallowing dynamics in real-time in pig airways, offering a promising non-invasive alternative solution for patients vulnerable to dysphagia.

• This research using an adjustable transducer probe capable of optimizing light path angles, enhancing the optical performance of photoacoustic imaging in real-time dysphagia.

**Supplementary Information:**

The online version contains supplementary material available at 10.1007/s44258-025-00062-6.

## Introduction

The ability to swallow food and drink is something that most of us take for granted, yet when it goes wrong, it can have disastrous consequences. Swallowing is also a remarkably complex behavior that requires reconfiguration of the upper airway from a pathway for breathing into a pathway for ingestion. Closure of the larynx, the entrance to the lower airway, is critical to prevent the aspiration of food, liquid, or saliva. Aspiration is a primary component of dysphagia (swallowing impairment), and it increases the risk of respiratory infection, morbidity, and mortality [1, 2]. Aspiration pneumonia has the highest attributable mortality of all medical complications post-stroke, or in individuals with progressive neurological diseases [3]. Dysphagia can occur across the lifespan, from premature infants to the very elderly, and has been estimated to affect 6.7% of hospital admissions in the United States [4, 5]. Recent studies also found that dysphagia significantly increases healthcare costs due to longer hospital stays and the need for specialized care [6, 7].

The clinical assessment of swallowing typically begins with a clinical “bedside” swallowing examination (CBSE), in which the clinician obtains patient history, performs an oral mechanism exam, and observes oral intake of different foods and liquids [8]. Aspiration typically leads to a cough response in healthy individuals. However, in patients with dysphagia, this cough response may be missing (“silent aspiration”), delayed, or ineffective in ejecting the aspirated material out of the airway [8]. As a result, the CBSE lacks both sensitivity and specificity for detecting aspiration [9, 10]. Instrumental swallowing assessments, involving either dynamic radiological exams -videofluoroscopy (VF), or Fiberoptic Endoscopic Evaluation of Swallowing (FEES), are widely accepted as “gold” standard approaches for dysphagia diagnosis [11]. Although these approaches allow for direct visualization of bolus flow and the presence of aspirated material in and below the larynx, these procedures have limitations. VF involves exposure to radiation, with a mean effective dose of approximately 1.23 mSv for a single exam (ten times higher than a chest X-ray), and must therefore be performed using a time-limited protocol [12]. Therefore, repeated VF exams are only warranted when a suspected change in status needs to be confirmed. The radiation from VF is particularly concerning for vulnerable populations, such as children, who are more sensitive to radiation [13–15]. The FEES exam requires the insertion of a nasal-endoscope, which can be uncomfortable; additionally, a brief period of white-out at the height of the swallow interrupts continuous viewing of the larynx, such that the occurrence of aspiration cannot be directly visualized, but is inferred based on the presence of residue in the larynx after the swallow [16].

To date, several new approaches for detecting swallowing and aspiration have been explored, including pulse oximetry [17], swallowing acoustics [18, 19], and swallowing accelerometry [20]. However, none of these technologies have demonstrated adequate sensitivity and specificity for detecting aspiration in comparison to the gold standards [21–23]. Recently, there has also been increasing interest in using ultrasound for dysphagia assessment, given that ultrasound is an established tool for imaging head and neck anatomy [24]. Pilot studies show that ultrasound can be used to assess hyoid displacement, which may be correlated with disordered swallowing [25, 26]. Ultrasound has also been used to visualize post-swallow residues based on areas of high echogenicity [27, 28]. However, a recent systematic review [29] pointed out that the sensitivity and specificity of these methods are very limited, and in general, ultrasound is not recommended as a tool to use in isolation. The limited performance of ultrasound is mainly due to (a) low contrast for residue detection, (b) signal degradation across tissue-air or fluid-air boundaries, and (c) operator-dependent transducer placement, leading to low reproducibility [29].

Therefore, there is a growing need for alternative diagnostic tools that offer safe, accurate, and non-invasive means of assessing swallowing functions. In recent years, photoacoustic imaging (PAI) has emerged as a promising technology in this realm [30, 31]. PAI, based on the photoacoustic effect, converts optical light absorption into acoustic energy, enabling high-resolution imaging of optical absorption in tissues [32, 33]. The nature of photoacoustic (PA) signal generation allows dark-colored food and liquids to be clearly visualized during the swallowing process [31].

Recently, we identified food-grade activated charcoal to exhibit even stronger absorption than conventional foods and liquids [34]. Our preliminary results indicate that a 10 mg/ml charcoal solution exhibited strong photoacoustic signals (~ 10 times higher than blood upon 1064 nm irradiation) when flowing into a phantom trachea. In this study, we validated the technique through ex-vivo pig experiments by successfully detecting the charcoal solution in the pig airway. Further, a preliminary in vivo experiment was conducted by integrating PAI with videofluoroscopy to monitor swallowing events. Our results indicate that PAI holds great potential in swallow evaluation and aspiration detection.

## Materials and methods

### Photoacoustic imaging system

**Fig. 1:**
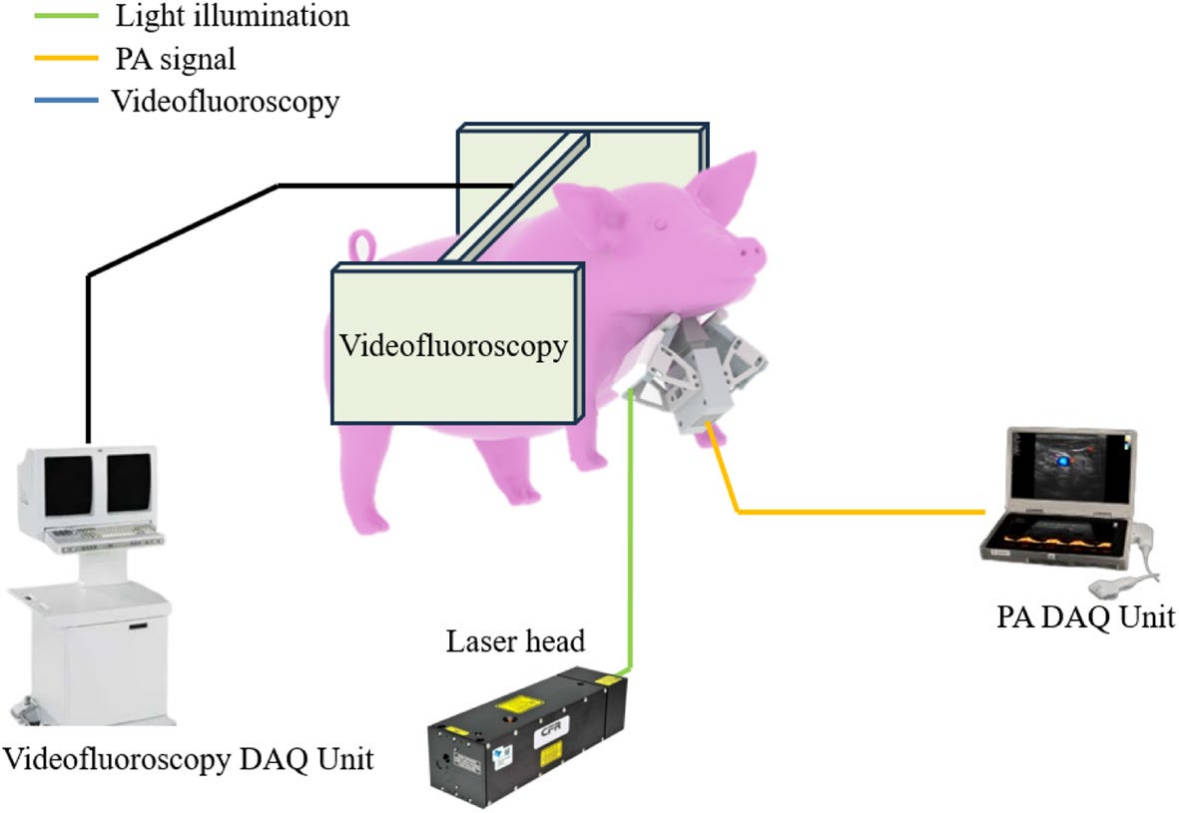
Schematic drawing of dual-modal PAI and videofluoroscopy system

**Fig. 2:**
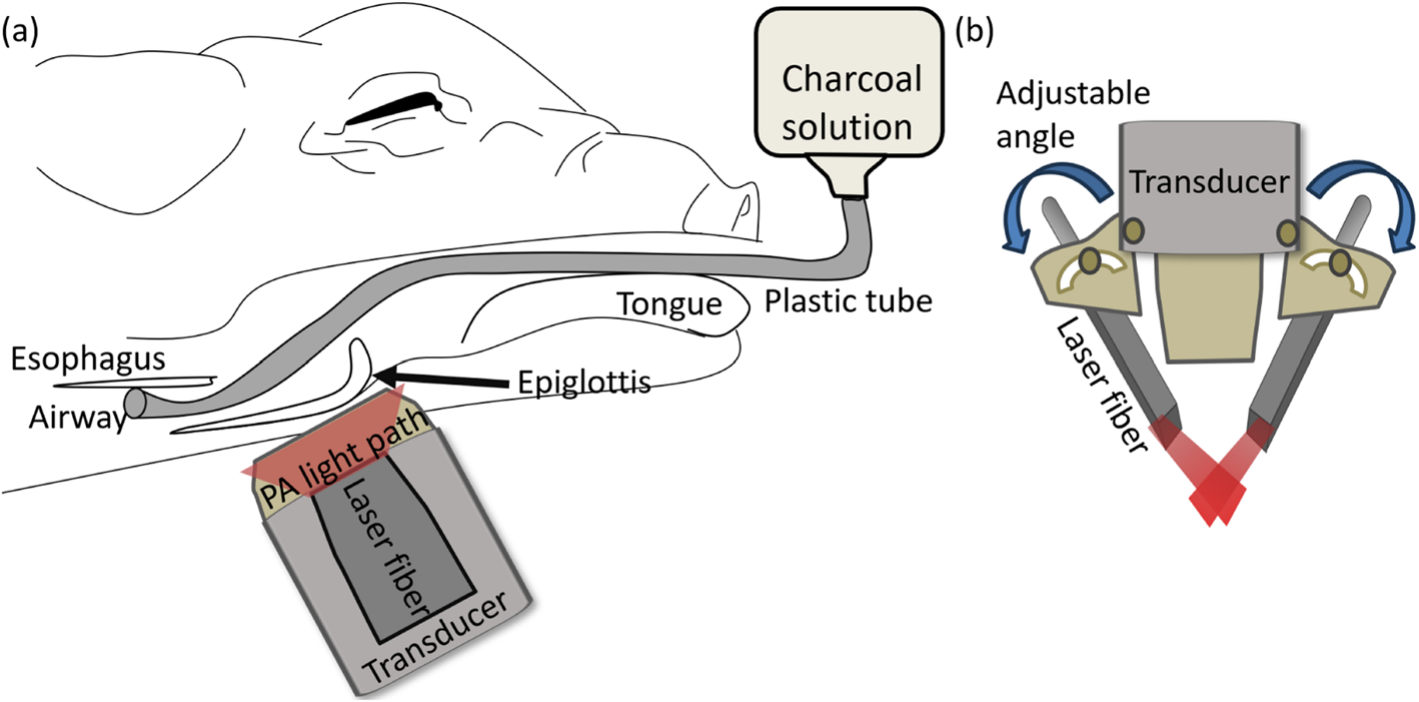
**a** Schematic drawing of PAI system and the pig anatomy in cross-sectional view. **b** Schematic drawing of the transducer probe combined with the adjustable light illumination

This study employed a compact PAI setup alongside the videofluoroscopy system as a dual imaging setup. As shown in Fig. 1, the setup comprises a portable ultrasound system (WinProbe, UltraVision Research Platform) coupled with a Nd:YAG laser (Bigsky laser, Quantel) [35]. The laser operates at a wavelength of 1,064 nm, a pulse repetition rate of 10 Hz, and a pulse width of about 8 ns. The PA signals were captured by a linear-array piezoelectric transducer (L7-4, Philips, The Netherlands) with a bandwidth of 4–7 MHz. It comprises 128 piezoelectric elements with a pitch distance of 0.28 mm and a total aperture size of 35.84 mm. The laser light illumination was provided by two linear fiber outputs mounted on the side of the transducer probe. As shown in Fig. 2, the optical fiber allows for easy adjustment of the focal zone to image different tissue depths. The WinProbe system features 64 transmit and receive channels, 14-bit digital resolution, 54 dB gain, and a sampling rate of 40 MHz. It captures signals at a 5 Hz frame rate due to the discrepancy between the number of transducer elements (128) and the number of Winprobe system receive channels (64). In the experiment, sterilized clear gel (AquaSonic Clear, Parker Laboratories, Inc.) was used to fill the gap between the transducer and the pig's neck skin.

This wavelength was chosen primarily for its ability to achieve deep tissue penetration and its low absorption by melanin [31, 36]. Furthermore, charcoal exhibits significantly higher absorption at 1064 nm compared to blood, enhancing image contrast and clarity [30]. Additionally, 1064 nm is the fundamental output of Nd:YAG lasers, which are more compact and portable than other laser systems, making them ideal for this application [37, 38].

The videofluoroscopy system was filmed in a lateral view while the infant pig drank the charcoal solution mixture in front of a fluoroscope (GE9400 C-Arm, 80 kV, 4MA). The filming was conducted using a high-speed, adjustable frame rate digital camera (XC 1 M digital video camera, Xcitex, Cambridge, MA) operating at 100 frames per second. This setup enabled real-time monitoring of the swallowing process in a side cross-sectional view, closely aligning with the cross-sectional perspective captured by photoacoustic imaging.

### Contrast solution preparation

The 10 mg/mL charcoal solution was chosen as the imaging contrast solution for its strong optical absorption and safety for ingestion. Based on our previous phantom study, this concentration provides strong signal intensity for effective tracking of the food movement in the airway [30]. We created the charcoal solution by mixing 250 ml of milk (Solustart Pig Milk Replacement, Land o’ Lakes, Arden Mills, MN), 250 ml of barium sulfate (E-Z Paque Barium sulfate, EZ EM Inc., NY), 1,000 ml of water, and 15 g of food-grade activated charcoal. This process resulted in a 1,500 ml mixture with a charcoal concentration of 10 mg/mL. The solution was thoroughly blended to ensure an even distribution of the charcoal.

### Ex vivo pig experiment

We first conducted photoacoustic imaging on pig cadavers to verify the PAI capability in detecting charcoal solution in the pig airway. For this experiment, we conducted PAI without videofluoroscopy, using 2 pigs with 2 scans performed on each. Before the experiment, the pig cadaver’s epiglottis was surgically modified to ensure unobstructed insertion of a plastic tube into the airway. Then, guided by a laryngoscope, we inserted a plastic tube into the trachea, allowing the charcoal solution to be delivered into the airway to mimic the aspiration events [39, 40].

To capture the dynamics of this process completely, we initiated PA imaging prior to the injection of the solution and continued imaging for a total of 60 s. This approach ensured that we recorded the airway's condition both before and after the introduction of the contrast solution. The injection rate was manually adjusted and took approximately 10 s to fully fill the entire airway. The angle of the optical fiber was adjusted to ensure optimum light fluence at the airyway. To optimize the imaging process, we adjusted the optical fiber angle during the ex-vivo experiment to align the laser’s focal zone with the aspiration site, typically around 10 mm beneath the skin. This ensured maximum light fluence at the target region, enhancing the detection capability of the PAI system. After identifying the optimal fiber angle in the ex vivo experiment, the same setup was used in the in vivo experiments to ensure consistent performance. As shown in Fig. 2(a), the PAI system could effectively capture signals of the charcoal solution within the airway.

### In vivo pig experiments

For the in-vivo study, we lesioned the pig’s internal Superior Laryngeal Nerve (iSLN) in a sterile surgery. This is based on our finding that a unilateral SLN lesion can change the temporal relationships among sucking and swallowing activities and increase the incidence of aspiration [25]. All procedures and data collection were performed under AAALAC standards and approved by the NEOMED Institutional Animal Care and Use Committees (IACUC) under protocol number #22–01-312.

**Fig. 3:**
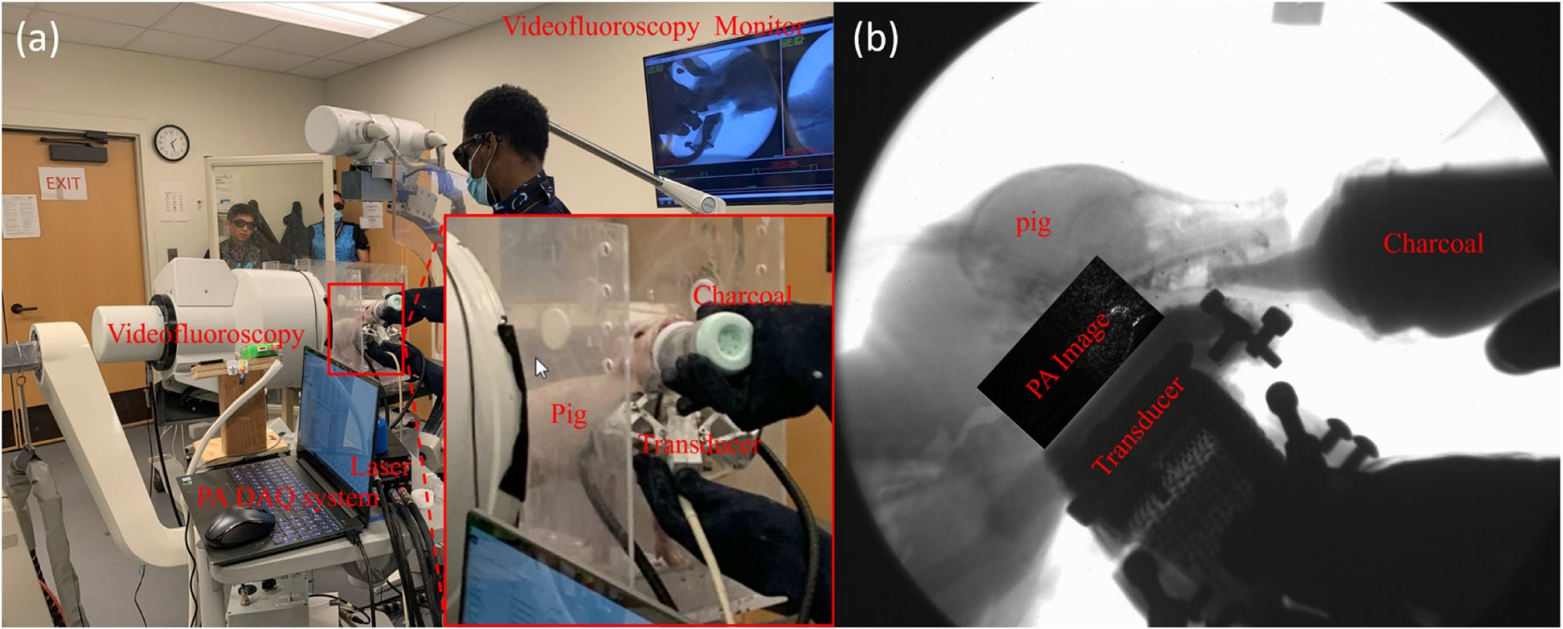
**a** A photo of the dual-modal system setup for live pig imaging. **b** One exemplary frame of PA image overlaid on X-ray imaging

The in-vivo PAI setup was identical to the ex-vivo experiment. In addition, we integrated videofluoroscopy imaging with PAI. During the imaging process, the ultrasound transducer was placed in front of the pig's neck, while the videofluoroscopy system imaged the pig from the side, as shown in Fig. 3(a). This arrangement allowed the two imaging systems to operate simultaneously without obstructing each other's field of view. Synchronization of the imaging modalities was achieved by manually starting the recording on each system simultaneously. By overlapping the videofluoroscopy imaging pathway with the photoacoustic imaging pathway, we can simultaneously visualize the movement of the contrast solution and the cross-section of tissue structures. Therefore, we can compare PA images directly to the videofluoroscopy result as shown in Fig. 3(b).

Additionally, a large wall-mounted monitor was installed right next to the imaging setup for real-time visualization of the videofluoroscopy images. This setup allowed us to adjust the ultrasound position in real time, which was crucial for ensuring the optimal positioning of the pig relative to the ultrasound transducer. An example of the PA image overlaid on the videofluoroscopy image is shown in Fig. 3(b), clearly displaying both the milk bottle and the ultrasound transducer within the image.

## Results

### Ex vivo imaging results

**Fig. 4:**
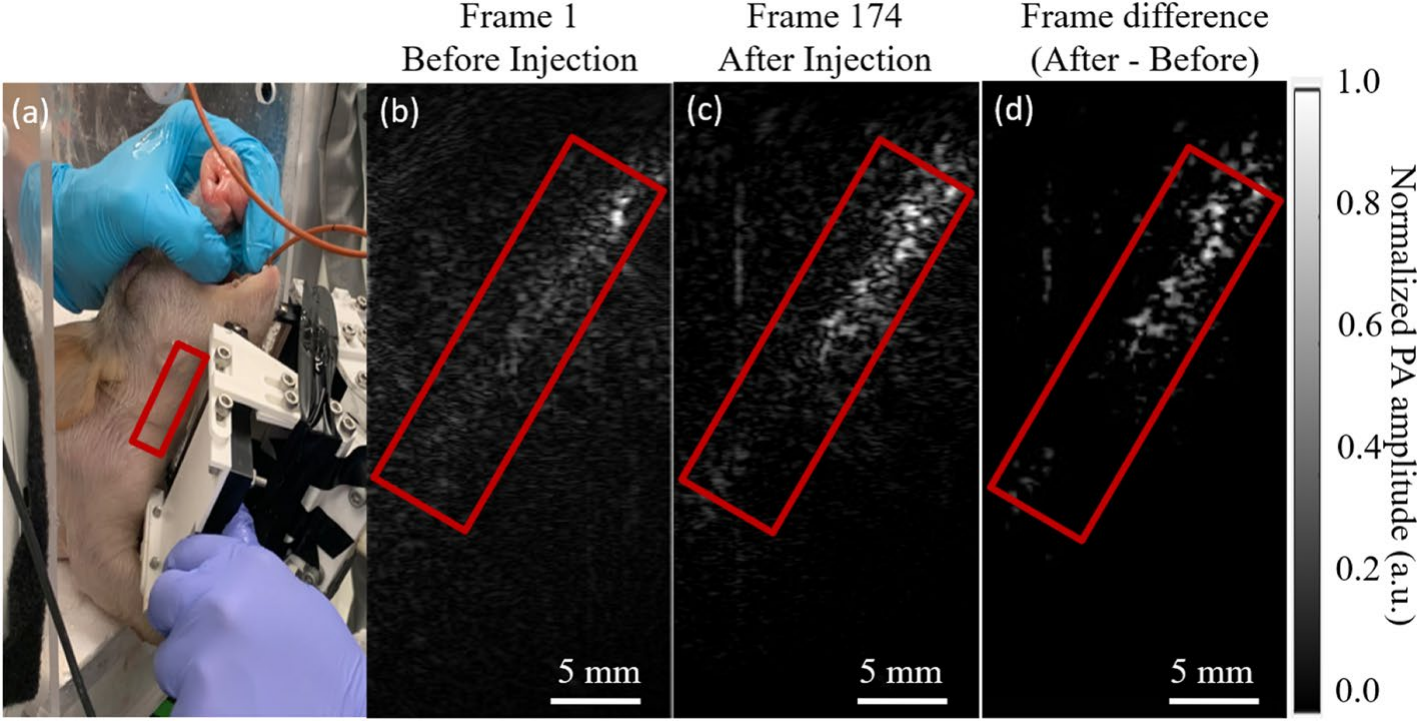
**a** A photo of the ex-vivo experiment setup. The red rectangle box shows the estimated position of the imaging area. **b** PAI images acquired **b** before and **c** after the charcoal injection **d** The differential image of before and after injection. PAI was performed at 5 frames per second

One representative result from ex vivo experiment is shown in Fig. 4. Figure 4(a) exhibits a photo of the imaging setup with the pig cadaver in the standing position and the plastic tube in the airway. The estimated imaging position is marked by a red rectangular box. Figure 4(b) shows PA image captured before the contrast injection. Due to native absorption from the plastic tube material, we observed weak photoacoustic signals along the airway. After contrast injection, the PA signals were significantly enhanced, as shown in Fig. 4(c), indicating the presence of the charcoal solution in the airway. The imaging depth ranges from 0 to 15 mm beneath the skin. Figure 4(d) presents a differential map highlighting significant increases in PA signals between pre- and post-charcoal injections, clearly visualizing the PA signal contribution.

### Dual-modal in vivo imaging results

**Fig. 5:**
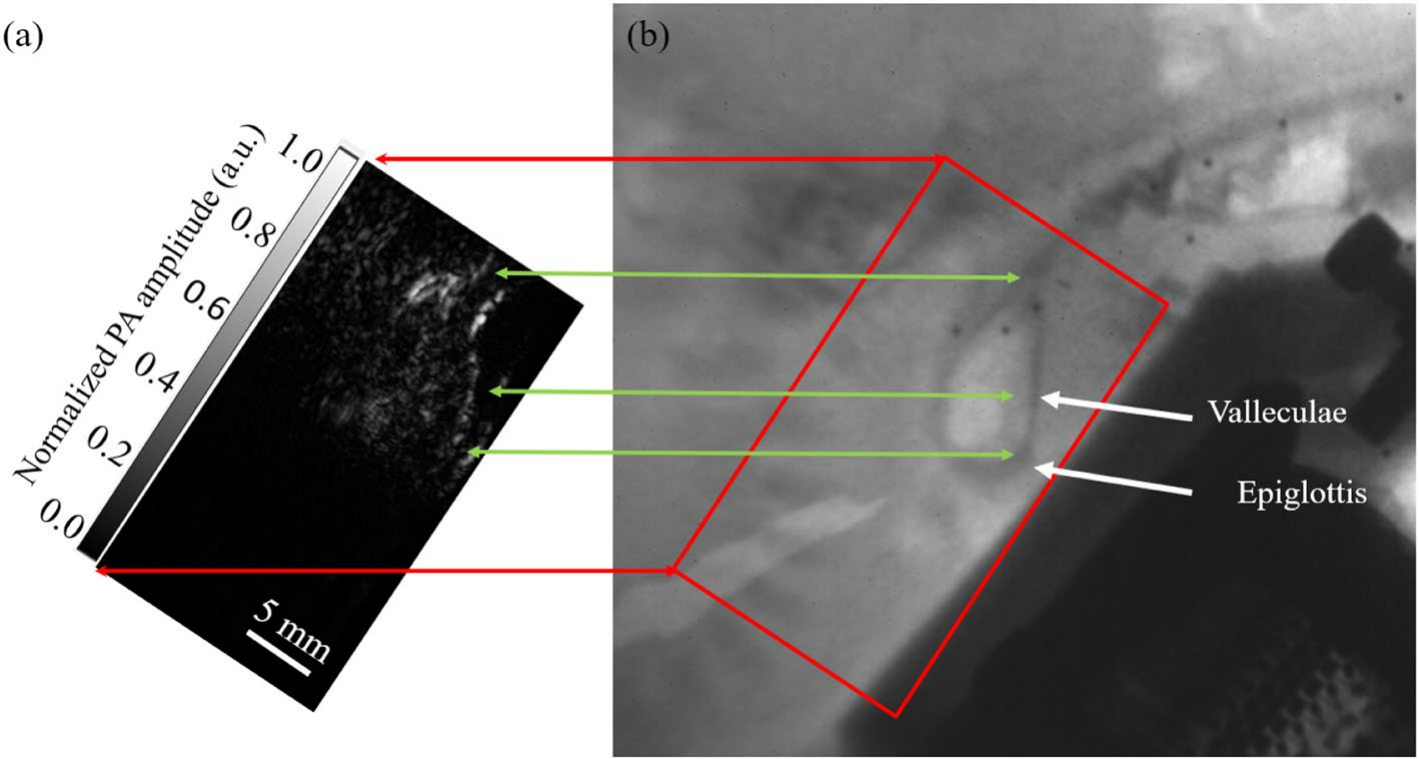
Side-by-side comparison of **a** PAI image and **b** the corresponding videofluoroscopy image. Similar structures are visualized in both modalities. PAI was performed at 5 frames per second. Videofluoroscopy was performed at 20 frames per second

In the in-vivo experiment, we used the same charcoal solution as in the cadaver study. One representative result is presented in Fig. 5(a), with the corresponding videofluoroscopy result shown in Fig. 5(b). The ultrasound transducer is clearly visible in the videofluoroscopy image, and the accumulation of contrast in the valleculae is distinctly observed [41, 42]. The PAI result revealed that the signal was strongest approximately at 10 mm depth beneath the skin. The estimated imaging overlap region is highlighted in a red rectangular box in Fig. 5, with the similar contrast structure indicated by a green arrow in both images. As the contrast shape aligns closely between the videofluoroscopy and PA image, we can confirm the successful capture of the swallowing process. However, a small misalignment between PAI and videofluoroscopy is observed, likely due to the imaging angles not being perfectly perpendicular to each other. Following the aspiration event, the pig's body moved, which led to the decoupling of the probe. Therefore, we were unable to continuously capture the moment immediately after the aspiration.

To validate our in vivo findings, we calculated the Structural Similarity Index Measure (SSIM) and other similarity metrics between the PAI and videofluoroscopy images. Given the differences in pixel values between the two imaging modalities, key features were extracted before conducting a quantitative comparison. We performed several steps, including thresholding, region of interest (ROI) selection, object resizing, and position matching, to ensure that features from the two modalities were co-registered. The quantitative analysis revealed a high degree of similarity in the charcoal solution's intensity within the airway, as shown in Table 1.

**Table 1:** Quantitative Analysis of Structural Similarity Between PAI and VF

Metric	Value	Metric	Value
SSIM	0.8918	NCC	0.3276

The SSIM of 0.8918 highlights aligned spatial features between the images. Additionally, the Normalized Cross-Correlation (NCC) of 0.3276 further indicates a good correlation in intensity patterns. The quantitative analysis collectively confirm that the two images share significant structural correspondence. The detailed image processing steps and metric calculations are provided in the supplementary material.

The live pig experiment was conducted under slightly different conditions from the cadaver study, but the PAI signals still exhibited a signal-to-noise ratio of 16.1 to the background tissue. Although the signal shape and distributions varied due to the dynamic nature of the live model, the overall ability of the PAI system to identify and distinguish the charcoal solution from other anatomical features remained consistent.

## Discussion

The PAI results from the in-vivo experiments demonstrated a good correlation with the videofluoroscopy imaging outcomes, effectively capturing heightened signal intensity from the charcoal solution during the swallowing process. Unlike videofluoroscopy, which exposes patients to ionizing radiation and poses cumulative exposure risks, PAI eliminates such concerns entirely. This makes PAI safer for vulnerable populations, including pediatric and elderly patients sensitive to radiation-related risks. Furthermore, PAI's high contrast sensitivity allows it to detect very small volumes of the charcoal solution, making it particularly effective for early and precise identification of swallowing events [43–46]. Our current efforts focus on validating PAI's capabilities through the integration and comparison of both techniques. The findings indicate that PAI shows great potential for swallowing assessment and aspiration detection.

However, while PAI shows considerable potential in swallowing process analysis, further experiments are necessary to fully validate its precision and effectiveness in reliably capturing aspiration events. The current limitations include the laser frequency, which only supports a low imaging capture rate, and transducer coupling consistency, which affects the stability of the acoustic connection during imaging [47, 48]. Specifically, the current PAI system operates at 5 Hz, which is much less than the frame rate of videofluoroscopy (20–100 Hz). This discrepancy hinders PAI’s ability to capture rapid aspiration events with the required detail. This can be addressed by using a higher pulse repetition rate laser.

On the other hand, maintaining consistent transducer coupling during in-vivo experiments is challenging, especially when the subject moves or coughs. In the future, we plan to implement motion compensation techniques, such as nonrigid registration methods, to mitigate motion artifacts and enhance image quality [49, 50]. Additionally, wearable probe designs can improve coupling stability, reducing issues associated with handheld transducers. Recent advancements in wearable ultrasound technology, such as flexible and stretchable devices, can provide continuous imaging capabilities [51–53]. Optimizing the probe into a wearable setup can address challenges associated with handheld transducers. Moreover, exploring the integration of VF and PA imaging may further enhance diagnostic accuracy by leveraging the complementary strengths of both modalities. These innovations are crucial for the reliable application of PAI in swallowing studies. Incorporating these strategies can significantly enhance the effectiveness of PAI in the future clinical settings.

Finally, our current study provides only a proof of concept investigation. In the future, additional live pig imaging experiments are needed to refine system setup and provide quantitative measures. These additional animal studies will help to move the system closer to human testing.

## Conclusion

Our study demonstrated the potential of PAI in monitoring swallowing events, with promising results in both pig cadaver and live pig experiments. The high optical contrast provided by PAI allowed for clear detection of the charcoal solution during the swallowing process, highlighting its capability to serve as a reliable alternative to videofluoroscopy for real-time dysphagia assessment. Implementing PAI in real-time dysphagia assessment could significantly enhance the early detection of aspiration events, thereby reducing the risk of aspiration pneumonia—a leading cause of morbidity and mortality in individuals with dysphagia [54–56]. Moreover, PAI's non-ionizing nature makes it safer for repeated use, particularly benefiting vulnerable populations such as infants and the elderly [57–59]. The portability of PAI devices also facilitates bedside assessments, improving patient comfort and accessibility to care.

To address the current limitations, future work will focus on enhancing the system’s frame rate and developing wearable probe designs to improve transducer coupling stability. Implementing advanced motion compensation algorithms will mitigate artifacts caused by patient movement, ensuring higher image quality. Additionally, integrating PAI with videofluoroscopy could combine the strengths of both modalities, further improving diagnostic accuracy and validation [60, 61]. In additional, future studies will also focus on increasing the sample size to obtain more reliable data and improve statistical significance. This will also enable a more quantitative measure of swallow dynamics and push the technique closer to clinical validation. The advancements anticipated from future studies will position PAI as a safer, non-invasive, and effective tool for dysphagia management, particularly benefiting vulnerable patient groups.

## Supplementary Information

Supplementary Material 1

Supplementary Material 2

Supplementary Material 3

## Data Availability

All data relatived with this study are present in the paper or the Supplemental Information. Raw data are available upon request.
