# Supplementary material for: Dysphagia assessment based on photoacoustic imaging: a pilot ex vivo and in vivo study in infant swine models
Source: Med X. 2025 Oct 22;3(1):19. doi: 10.1007/s44258-025-00062-6 (PMC12546401; doi:10.1007/s44258-025-00062-6)
Supplement: Supplementary file 3 — Supplementary Material 3 [file 44258_2025_62_MOESM3_ESM.docx]

Supplementary Materials for **Dysphagia assessment based on photoacoustic imaging: a pilot *ex vivo* and *in vivo* study in infant swine models**

Video 1: Slow Motion PA Imaging Video Demonstrating the Swallow Process

Video 2: Corresponding Slow Motion VF Imaging Video


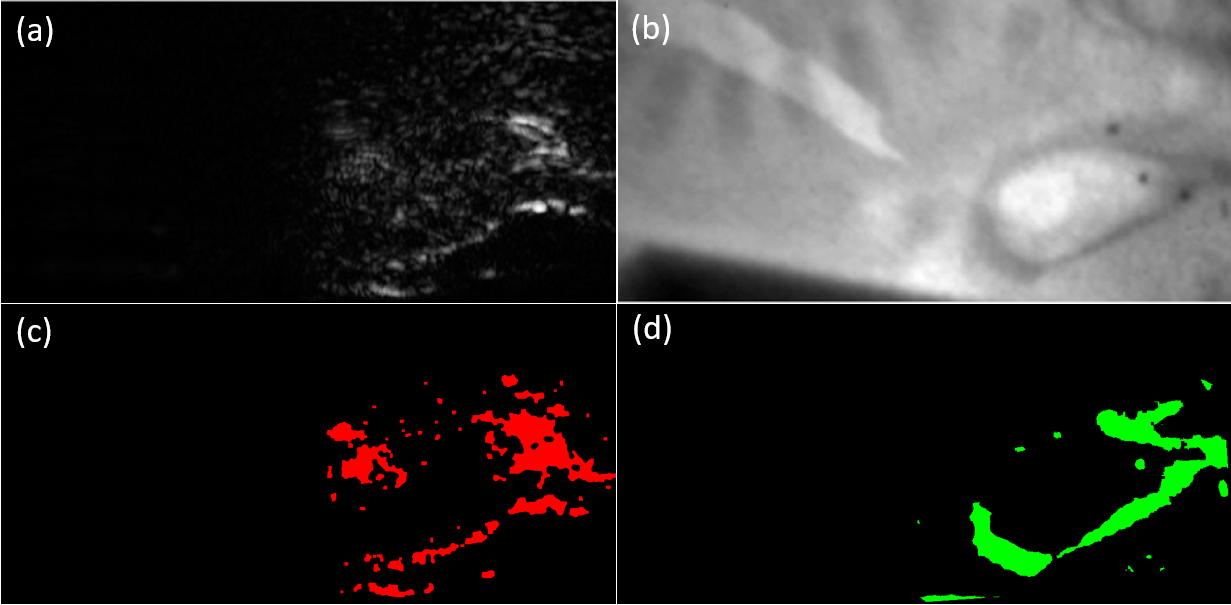


Figure 1: Similarity comparison: (a) PA image, (b) Corresponding VF image with the same region of interest as PA, (c) Processed PA image, (d) Processed VF image.
